# Female-specific pharmacotherapy in ADHD: premenstrual adjustment of psychostimulant dosage

**DOI:** 10.1192/j.eurpsy.2025.224

**Published:** 2025-08-26

**Authors:** M. De Jong, D. S. Wynchank

**Affiliations:** 1 Expertise Centre Adult ADHD, PsyQ, The Hague; 2 VU Medical Centre, Amsterdam Public Health Research Institute; 3 Department of Psychiatry, Amsterdam UMC/VUmc, Amsterdam, Netherlands

## Abstract

**Abstract: Objective:**

Attention-Deficit/Hyperactivity Disorder (ADHD) is a common neurodevelopmental condition which is underdiagnosed and undertreated in women. For decades, the ADHD field has called for more insight into female-specific therapy. Preliminary findings postulate that changes in sex hormones during the menstrual cycle may influence the effectiveness of psychostimulant medication. Yet, pharmacotherapeutic interventions tailored to women with ADHD remain scarce. Previously, our group showed an increase in mood symptoms in the premenstrual week in women with ADHD. Premenstrual worsening of depressive and ADHD symptoms represent a treatment challenge.

In our adult ADHD clinic, we noted several women describing exacerbation of their ADHD and depressive symptoms in the premenstrual week and/or insufficient effect of their established dosage of psychostimulant. We responded to the need expressed by these women by increasing their stimulant dosage in the premenstrual week, while monitoring the response and side effects.

**Methods:**

This community case study of nine consecutive women being treated for ADHD and co-occurring conditions (including depression and premenstrual dysphoric disorder), reports our local experience of increasing the individually prescribed psychostimulant dosage during the premenstrual period. We methodically monitored the effect of this increased dosage on ADHD symptoms, mood and somatic symptoms for the following 6-24 months.

**Results:**

With premenstrual dose elevation, all nine women experienced improved ADHD and mood symptoms with minimal adverse events. Premenstrual inattention, irritability and energy levels improved, and now resembled the other non-premenstrual weeks more closely. All women decided to continue with the elevated premenstrual pharmacotherapy.

**Discussion:**

Our preliminary results demonstrate potential benefits of increasing premenstrual psychostimulant dosage in women with ADHD, experiencing premenstrual worsening of ADHD and mood symptoms. The results concur with previous findings of diminished response to amphetamines in the late luteal phase. Increased dosage may help combat premenstrual worsening of cognitive and emotional symptoms in women with ADHD, with significant clinical implications. Better management of premenstrual ADHD and mood symptoms in vulnerable women can improve treatment outcome and meet an unmet need. However, implementation should be individually explored. Further investigation of luteal phase psychostimulant dose adjustment is required for safe, optimal and individualised treatment for women with ADHD. Specifically, we are currently applying for funding to set up the ADAPT-trial (Adjusting Premenstrual Psychostimulant Dosage for Women with ADHD: Enhancing Efficacy).

**Disclosure of Interest:**

None Declared

